# Cohort Profile Update: The Amirkola Health and Ageing Project (AHAP)

**DOI:** 10.22088/cjim.8.3.205

**Published:** 2017

**Authors:** Ali Bijani, Reza Ghadimi, Ebrahim Mikaniki, Farzan Kheirkhah, Seyyed Ali Mozaffarpur, Mina Motallebnejad, Haleh Esmaili, Fatemeh Majidi, Robert Graham Cumming, Seyed Reza Hosseini

**Affiliations:** 1 Social Determinants of Health Research Center, Health Research Institute, Babol University of Medical Sciences, Babol, Iran.; 2 Traditional Medicine and History of Medical Sciences Research Center, Health Research Institute, Babol University of Medical Sciences, Babol, Iran.; 3 Oral Health Research Center, Health Reseach Institute, Babol University of Medical Sciences, Babol, Iran.; 4 Health Research Institute, Babol University of Medical Sciences, Babol, Iran.; 5 *Cellular* & Molecular Biology *Research Center,* Health Research Institute, Babol University of Medical Sciences, Babol, Iran.; 6 School of Public Health, University of Sydney, Sydney, Australia.

**Keywords:** Older People, Cohort, Ageing, Health, Iran

## Abstract

The original cohort study of AHAP started in 2011 on 1616 elderly residents of Amirkola, northern part of Iran near the Caspian Sea. The main goal of this study was to comprehensively evaluate the health of the elderly in the region with the emphasis on chronic diseases such as osteoporosis. The first cohort profile was published in the International Journal of Epidemiology in 2014. The phase 1 AHAP showed the elevated level of some diseases and conditions including osteoporosis, metabolic syndrome, obesity, vision problems and relatively low levels of oral health. Therefore, the second phase of this cohort started with more complete population coverage in 2016, not only to collect and record the information based on previous protocol, but also consider new areas such as nutritional status, complete eye and dental examinations and health assessment on the basis of Iranian Traditional Medicine. The new aspect of this project is to conduct *clinical and laboratory examinations at the health center to extend *more facilities to the elderly. In addition to serum and DNA, samples of saliva, hair and nails are collected and kept under standard conditions in the biobank of this cohort. Researchers can apply for access to data or suggest a collaborative study by submitting the proposal to AHAP committee.

## The original cohort

The Amirkola Health and Ageing Project (AHAP) is the first *comprehensive cohort* project of the elderly in Iran (1) that composed 1616 elderly people in 2011-2012. Amirkola is a small and middle-class income town with low immigration rate and a suitable model of a city for cohort studies. Since exposure is identified before the outcome, cohort studies are appropriate to demonstrate causality and are the best choice for the calculation of incidence rate and cumulative incidence of diseases or health outcomes, especially the chronic diseases (2, 3). Amirkola cohort study was designed with regard to multiple chronic diseases and health concern like as osteoporosis, cognitive impairment and fall. 

The strengths of the study are the different exposures and the *adequate coverage* of the *population *of which 72.3% of the elderly aged 60 years and over in Amirkola were entered into the study. Besides the various hormonal and biochemical tests, their serum and DNA samples are maintained in standard conditions for future research. Filling out a questionnaire and preliminary examination were carried out phase in 1 performing project center and the elderly were referred to specialty centers for ophthalmic, psychiatric, internal and bone densitometry examinations (1). 

## What is the reason for the new focus and new data collection?

The first phase of AHAP study comprising 72.3% of Amirkola's aging population was conducted by home visits after sending two invitation letters, and sampling and data recording of more than 10% of them, were done at home. Then, they were referred to the other centers outside this city for densitometry and other specialized examinations which led to the loss of 25% elderly people (1). Therefore, the integration of the laboratory and clinical examinations seemed necessary for the elderly to provide them additional facilities by setting a new space for collecting data on more favorable situations. On the other hand, the obtained results from more than 50 published articles of this project indicated the high prevalence of certain diseases and health problems in the region, which was unexpected before the beginning of the project (4). 

Table 1 summarizes some findings of AHAP phase I. These results showed high prevalence of metabolic syndrome (more than 70%), especially in women (5), has emphasized the need for assessment of nutritional status of the elderly.

**Table 1:** Some main findings of AHAP phase I

**First author [year]**	**Study information**	**Main results**
Bayani MA [2016] (5)	Subject: Metabolic syndrome N= 1562 (M=865, F=703)	High prevalence of metabolic syndrome (74.3%) in males: 65%, in females: 85.7%
Rasoulinejad SA [2015] (6)	Subject: Hypertensive retinopathy N=505 (Patients referred to the eye clinic)	Prevalence of hypertensive retinopathy: 12.9%
Rasoulinejad SA [2015] (7)	Subject: Age-related macular degenerations (AMD) N=505 (Patients referred to the eye clinic)	Prevalence of AMD: 17.6% Correlation between AMD and smoking: Adjusted OR:5.03 (CI95%: 2.47-10.23)
Hosseini SR [2013](8, 9)	Subject: Oral health	Mean decay-missing-filled (DMF) = 25.33±8.96 Significant association between DMF with smoking and depression
Hosseini SR [2014](10)	Subject: Bone mineral density (BMD) N=1000 (M=554, F=446)	Positive association between body mass index (BMI) and BMD Pearson correlation between BMI and BMD T.score: Lumbar spine: r=0.16, p<0.001 Femoral neck: r=0.35, p<0.001
Heidari B [2015](11)	Subject: Osteoporosis (OP) N=537 (Post-menopausal women)	Significant association between OP with menopause duration, parity and history of fracture Significant negative relationship between OP with metabolic syndrome, obesity, serum ferritin, physical activity and high educational level
Heidari B[2017](12)	Subject: Osteoporosis (OP) N=553 (Males)	Prevalence of OP:16.2% Positive association between OP with age, anemia and prior fracture Negative association between OP with obesity, muscle strength and diabetes
Teimorian M[2016] (13)	Subject: Osteoporosis N= 156 (82 osteoporotic and 74 healthy individuals)	Increasingimmune hormone IL-10 and parathormone in the serum of osteoporosis vs. non-osteoporosis people **(p<0.05)**
Porhashem Z [2012] (14), Ghadimi R[2017] (15), Mirzapour A [2016] (16), Bayani MA [2016] (17), Seyfizadeh N[2016](18)	Subject: Osteoporosis (OP)	Association between low mineral mass and smoking. Higher mean of lumbar and femur bone mineral densities in older people with diabetes than without diabetes. Significant direct correlation between normal levels of TSH and femoral density in females but not in males No significant association between OP with serum vitamin D level, RH and ABO blood types
Ghasemi A [2015](19)	Subject: Diabetes & Serum prostate-specific antigen (PSA) levels N= 792 Males	Mean PSA levels: 1.88±2.98 Significant correlation between PSA and BMI (p=0.001). No association between PSA levels and diabetes.
Mirzapour A [2015](20)	Subject: Diabetes N=1525	Prevalence of diabetes: 30.6% (known diabetes: 23%, undiagnosed diabetes: 7.6%) Inverse relationship between FBS and serum uric acid level (r=-0.054, p=0.012).
Moudi E [2017](21)	Subject: Nephrolithiasis N=1390 (M=779, F=611)	Prevalence of Nephrolithiasis: 14.53% (in males: 16.8%, in females: 11.6%) Association between nephrolithiasis with male gender, obesity and age<75y
Moudi E [2016](22)	Subject: Microscopic hematuria N=1243 (M=674, F= 596)	Relationship between microscopic hematuria and aspirin use (adjusted OR=1.40, CI95%: 1.02-1.92)
Moudi E [2017] (23)	Subject: Urinary incontinence N=590 (Females)	Prevalence of urinary incontinence: 32.9% Increasing the possibility of the urinary incontinence with constipation history, spouseless status and use of corticosteroid drugs
Bijani A [2014] (24)	Subject: Drugs N=1534 (M=842, F=692)	Prevalence of polypharmacy (in females: 35.12%, in males: 16.51%, p<0.001)
MonemiAmiri A [2015] (25), Hosseini SR [2015] (26), Taghipour M [2016] (27)	Subject: Physical activity	Low level of physical activity in older people. Significant relationship between physical activity with bone mineral density, balance control, etc. Significant inverse correlation betweenphysical activity with age, drug use and chronic diseases
Hosseini SR [2016](28) Vakili Sadeghi M [2017] (29)	Subject: Falls N=1616, N=1482	Prevalence of falls (the last 12 months): 17% (in males: 14%, in females: 22.4%, p<0.001). No relationship between falls with serum vitamin D levels and anemia
Alipour M[2015](30), *Taghipour-Darzi M*[2013] (31)	Subject: Chronic musculoskeletal pain N=857 (M=599, F=258) & N= 1614 (M=883, F=731)	Prevalence of chronic musculoskeletal pain: (77.7%-82.4%) The most common sites of pain: the knees, back, feet and shoulders No relationship between chronic musculoskeletal pain with obesity and female gender No significant relationship between chronic musculoskeletal pain with vitamin D level *and functional disability*
Babaei M [2017](32)	**Subject: Iron deficiency anemia (IDA)** **N=240 (IDA+: 80, IDA-: 160)**	**Ability of serum ferritin to diagnose** IDA (AUC=0.615, CI95%: 0.536-0.694)
Sotuneh N [2014] (33), Fotouk-kiai[2015] (34)	Subject: Helicobacter pylori (HP) N=1300 (M=759, F=541) N=967	Prevalence of HP infection: 76.2% (in males: 78.3%, in females: 73.4%) No significant relationship between the level of antibodies against HP with bone mineral density (BMD), BMI, FBS and lipid profiles.
Mouodi S [2016](35)	Subject: Living alone N= 1544 (M=841, F=703)	Frequency of living alone: %6.8% Significant relationship between living alone and many disorders or disabilities (fall, symptoms of depression, unexplained headaches, etc.)
Ahmadiahangar A[2016] (36)	Subject: Headache N=1499 (M=832, F=667)	Prevalence of headache: 42% Significant dose-response relationship between depression and headache: Mild depression: OR:2.59 (CI 95%: 2.03-3.31) Moderate depression: OR:3.65 (CI 95%: 2.58-5.1) Severe depression: OR:6.04 (CI 95%: 3.54-10.3)
Kheirkhah F [2014] (37), Ahmadiahangar A[2014](38), Rezaeimehr Z [2016] (39), Kheirkhah F[2016](40)	Subject: Cognitive disorders	Prevalence of cognitive **impairment**: 18.3%- 31.5% Some related factors with cognitive **impairment**: Age, low educational level, spouseless, female gender, lived lonely, low bone mineral density, etc. **No **significant **association between **cognitive disorders **with serum vitamin D levels**, H. pylori infection, etc.
Faramarzi M [2017] (41), Kheirkhah F [2016] (42), Faramarzi M [2015] (43), Kheirkhah F [2015] (44)	Subject: Depressive disorders	Prevalence of depressive symptoms: 43.4% Some protective factors against depression: Social support, occupation and high educational level, etc. Some related factors for depression: Cognitive impairment, female gender, chronic pain, comorbidities, smoking, spouseless, low testosterone levels (in males), etc.
Younesi S[2015] (45), Kheirkhah F [2017] (46), Kheirkhah F [2016] (47), Negahdar H [2015] (48), Moallem M [2016] (49), Momeni H [2015] (50), Gezel S**[**2016] (51), Aleahmad A [2015](52)	Subject: Relationship between some trace elements and some disorders.	The relationship between some elements (copper, zinc, selenium, manganese and others) and oxidant/antioxidant states with some diseases (osteoporosis, depression, cognitive disorders, physical disabilities and others) in these studies

The high incidence of low vision in initial tests and self-report ocular complications in the older people and the referred elderly to the Clinic of Ophthalmology (6, 7) cause to consider a new phase of comprehensive medical eye examination for all ageing elderly, not only those with clear problems but also in the presence of an ophthalmologist in the data collection center. Their relatively oral health poor condition (8, 9) justifies the dental examinations. Since standard biobank is very important in most cohort projects of the world and their role is undeniable and due to the experiences of AHAP phase I, the storage of hair, fingernails and saliva samples plus serum and DNA have been considered and emphasized. 

In recent decades, Iranian medical education policy makers have planned to consider Iranian Traditional Medicine (ITM) by establishing of faculties of Traditional Medicine in Medical Sciences Universities and adaptive studies of a combination of traditional and modern medicine. Because of interest to ITM among the average population of the northern community, especially the elderly people, collecting the index of ITM and results of examinations of the elderly are considered in the phase II of AHAP project. 

## What will be the new area of research?

Although maintaining the conceptual basis of the cohort, identified exposures and outcomes of the original cohort is the most important part of this study, the possible integration of previous specialized and laboratory examinations such as densitometry and psychiatric examinations in centralized physical space and biobank promotion are the two new approaches in phase II. The second phase of the cohort includes new four areas: comprehensive nutritional assessment, medical eye examination, dental examination and elderly assessment from the perspective of ITM.

## Who is in the cohort?

Sampling of AHAP phase II commenced in 2016. All 1616 elderly people of phase I of AHAP cohort were followed-up. The exact date of death, place (home, hospital and etc.) and causes of death were recorded during his/her participation. Moreover, the others were entered into the study with the specified timing based on the list and most of their information up to now has been recorded similar to phase I. After two invitations, collecting of data and sampling from *disabled people *were done in their homes, which are now going on. Furthermore, the elderly living in Amirkola but could not enter to the previous phase for any reason or recently have migrated to this region or new participant who have surpassed exceeded 60 years since 2012 are invited to enroll in the study. 

So far, more than 1,700 clinical and laboratory examinations of elderly people have been recorded and it has been anticipated that by the end of this phase, this number exceeds 2,000. It seems that the loss of elderly people in phase II for reasons other than death was under 5%. Participants completed the informed consent form and this project was approved by the Research Ethics Committee of Babol University of Medical Sciences.

## What has been measured?

To provide more facilities for the elderly population, the project of this phase has been performed on the ground floor of a building on the main street of Amirkola, by the Social Determinants of Health Research Center of Babol University of Medical Sciences and most of the data on the elderly (except the advanced diagnostic and therapeutic referrals) are collected in this center. With same state as the first phase, filling out some parts of preliminary non-specialized questionnaires takes around 45 minutes at home. On top of the gathered information in phase I of the original cohort profile (1), new cases including questionnaires, clinical examinations and tests and changes in some procedures are shown in table 2.

In this phase, telephone follow-up will be continued. In new areas of phase 2, dental examination and information are recorded by a dentist, likewise, all ophthalmology information using slit lamp, non-contact tonometer, direct and indirect ophthalmoscopy and retinoscopy is recorded by an opthalmologist.

Elderly dietary information has been collected using a designed 138 items Semi-Quantitative food Frequency questionnaire (SQFFQ) based on commonly consumed food in this region in elderly. The reliability and validity of this questionnaire were evaluated on 200 elderly people using two *24*-*hour* dietary *recall questionnaire* (24H-RQ) and some *anthropometric* and *laboratory parameters*. Dietary information has been gathered by trained researchers through interviews with elderly people and their relatives.

Bone mineral density is measured by dual-energy x-ray absorptiometry (DEXA) method using horizon Wi (Hologic Company, USA) of in research center. Additionally, bone density of wrist, whole-body scan and soft tissue density including total, regional and visceral fat besides the hip and spine densitometry are determined. From each elderly individual, 25 cc blood, 2 cc saliva, urine sample, nails of both hands (one week from the last nail clipping) and about 1cm^2^ (some if not the exact amount) hair close to the root of the occipital region are taken. Since adult population-based cohort of Iran (PERSIAN cohort) (61) has been planned widely in the country since 2014, preparing the biobank and maintenance of the data in the current study are almost similar to the standards of that study to compare and share the data of AHAP with mentioned cohort (1, 61 , 62).

**Table 2:** New measurements in the Amirkola Health and Ageing Project (AHAP)

**Measures**	**Instruments **
**Questionnaires**	
Frailty Sleep Incontinence Knee osteoarthritis Nutrition Dental Traditional Medicine	The Simple “FRAIL” Questionnaire Screening Tool (53, 54) The Pittsburgh Sleep Quality Index (55) The 3 incontinent questions (3IQ) (56, 57) American College of Rheumatology criteria for the diagnosis of knee osteoarthritis (58) ***Semi-Quantitative Food Frequency Questionnaire (SQFFQ)*** Persian version of the General Oral Health Assessment Index (GOHAI)(59) Mojahedi's ten-item Mizaj questionnaire (60)
**Examinations**	
Eye Dental Balance Gait	Complete Eye Examination by Ophthalmologists By dental specialists Tandem gait, semi tandem 3-meter walk
**Blood tests**	
Routine biochemistry and Haematology	ALP, ALT,AST, Albumin, CRP
**Samples**	
Nail Hair Saliva	Nails of both hands (so that one week passes from the last nail clipping) Hair samples from occipital region were cut as close to the scalp as possible. (1cm^2^) *Unstimulated saliva*(1-3ml)

## What are the main strengths and weaknesses?

The strengths of the current study are (I) high participation rate (II) middle-class level and relatively stable population, (III) free clinical and laboratory services in which we build participants' trust (IV) centralized medical examinations and laboratory test (V) expert research team including more than 50 medical professionals and (VI) comprehensiveness of the project in the elderly population with evaluation of different exposures and outcomes.

The weaknesses of the present study are (I) the low level of education of the elderly (II), the inability to collect complete details of the elderly with severe disabilities, and (III) the loss of some useful information such as details of new outcomes between different phases of the cohort due to the lack of full registration system along with limitations of financial statement every two years in the region. It seems that the middle-age population coverage (45-60y) with larger size and the limited variables can improve this project.

## Can I get hold of the data? Where can I find more information?

Researchers can apply for access to data or suggest a collaboration study by submitting the proposal to AHAP committee in SDA research center of Babol University of Medical Sciences. For more information, please contact Prof. SR. Hosseini, the person in charge of this project (Email address: hosseinirezaseyed@gmail.com).
